# Comprehensive evaluation system for vegetation ecological quality: a case study of Sichuan ecological protection redline areas

**DOI:** 10.3389/fpls.2023.1178485

**Published:** 2023-06-26

**Authors:** Linlin Cui, Yanhui Chen, Yue Yuan, Yi Luo, Shiqi Huang, Guosheng Li

**Affiliations:** ^1^College of Resources and Environment, Chengdu University of Information Technology, Chengdu, China; ^2^Institute of Geographic Sciences and Natural Resources Research, Chinese Academy of Sciences, Beijing, China; ^3^College of Tourism and Geographical Science, Jilin Normal University, Siping, China; ^4^Sichuan Meteorological Disaster Prevention Technology Center, Sichuan Provincial Meteorological Service, Chengdu, China

**Keywords:** vegetation ecological quality, climate change, human activities, ecological protection redline, remote sensing

## Abstract

Dynamic monitoring and evaluation of vegetation ecological quality (VEQ) is indispensable for ecological environment management and sustainable development. Single-indicator methods that have been widely used may cause biased results due to neglect of the variety of vegetation ecological elements. We developed the vegetation ecological quality index (VEQI) by coupling vegetation structure (vegetation cover) and function (carbon sequestration, water conservation, soil retention, and biodiversity maintenance) indicators. The changing characteristics of VEQ and the relative contribution of driving factors in the ecological protection redline areas in Sichuan Province (EPRA), China, from 2000 to 2021 were explored using VEQI, Sen’s slope, Mann-Kendall test, Hurst index, and residual analysis based on the XGBoost (Extreme gradient boosting regressor). The results showed that the VEQ in the EPRA has improved over the 22-year study period, but this trend may be unsustainable in the future. Temperature was the most influential climate factor. And human activities were the dominant factor with a relative contribution of 78.57% to VEQ changes. This study provides ideas for assessing ecological restoration in other regions, and can provide guidance for ecosystem management and conservation.

## 1 Introduction

As a bearer and feeder of climate change, vegetation is an essential type of surface landscape cover and an important part of the global ecosystem. Moreover, it also has a vital role in water purification, soil conservation, water conservation, climate regulation, carbon sequestration, and biodiversity conservation (Peteet, 2000; Kong et al., 2018; Cui et al., 2022). Changes in the vegetation ecological quality (VEQ) reflect regional or global environmental changes and affect ecosystem functions. Climate change and unchecked exploitation have led to serious ecological problems related to vegetation, such as degradation of grasslands, land desertification, low-quality forest systems, pure forest structure, and inefficient forest functions (Wang et al., 2017; García et al., 2020). Therefore, monitoring dynamic changes of VEQ and quantifying the contribution of its main influencing factors are critical to promote ecological conservation and sustainable development.

VEQ refers to the similarity and relative integrity of vegetation structure, the lushness of the growth state, and the strength of its ecological service function compared with natural vegetation at the reference site. There are two significant groups of VEQ evaluation methods: field survey-based methods and remote sensing-based methods. The first methods (e.g., ecological conservatism coefficient and FQAI method) make use of field surveys to obtain vegetation quality attribute data (e.g., plant species, cover, and height) (Sanaei et al., 2019; Zinnen et al., 2021; Haq et al., 2022), which is not only time-consuming and laboriously inefficient but also problematic in terms of steadily translating site data to regional or global scales, and making it challenging to achieve long-term dynamic monitoring. In contrast, remote sensing technology has the advantage of a wide observation range, fast information acquisition, and short update cycles. The improvement of remote sensing data quality and the development of remote sensing mapping and quantitative inversion techniques also provide strong support for acquiring vegetation ecological characteristics. This makes remote sensing data helpful in assessing VEQ (Skidmore et al., 2010; Bajocco et al., 2012; Salvati and Ferrara, 2013; Trevisan et al., 2020; Poornazari et al., 2021). Currently, remote sensing evaluation of VEQ mainly uses a single indicator of vegetation ecological structure or function (e.g., vegetation cover, biomass, biochemical components, landscape index) (Qian et al., 2020). Vegetation ecosystems are complex, and the evaluation of VEQ is multi-dimensional. A single indicator can only reflect the change characteristics of a specific dimension of vegetation ecology, which may cause biased results (Li C. et al., 2022). Therefore, it is necessary to propose a VEQ evaluation method that couples multiple vegetation ecological indicators.

Climate change and human activities are the primary drivers of changes in VEQ. Vegetation dynamics and their response to climate change and human activities differ significantly due to regional differences in hydrothermal conditions, topography, soil, vegetation types, and human activity intensity (Fang et al., 2002; Peng et al., 2019). Numerous studies have analyzed vegetation changes using NDVI (normalized difference vegetation index) or NPP (net primary productivity) time-series data and quantified the relative contribution of influence factors (Leroux et al., 2017; Ge et al., 2021; Yu et al., 2021). The residual analysis based on multiple linear regression is widely used to quantify the relative contribution. Numerous studies have shown that the effects of environmental variables on vegetation are non-linear (Piao et al., 2015; Chen et al., 2019; Yang et al., 2021). Therefore, the residual analysis method still needs to be improved to satisfy the nonlinear relationship between vegetation change and environmental variables. XGBoost (eXtreme Gradient Boosting) is an integrated learning algorithm that is fast, accurate, robust, and performs well in dealing with nonlinear problems (Ma et al., 2020). The residual analysis method based on the XGBoost for quantifying the relative contribution of climate change and human activities to vegetation change may be a better idea.

Based on the above problems, the objectives of this study are: (1) to develop an vegetation ecological quality index (VEQI) by coupling multiple vegetation ecological elements; (2) to analyze the Spatio-temporal changes in VEQ from 2000 to 2021 using the ecological protection redline areas in Sichuan Province (EPRA) as the case study area; (3) to construct a residual analysis method based on the XGBoost to quantify the relative contributions of climate change and human activities to changes in VEQ.

## 2 Data and methods

### 2.1 Study area

**Figure 1:**
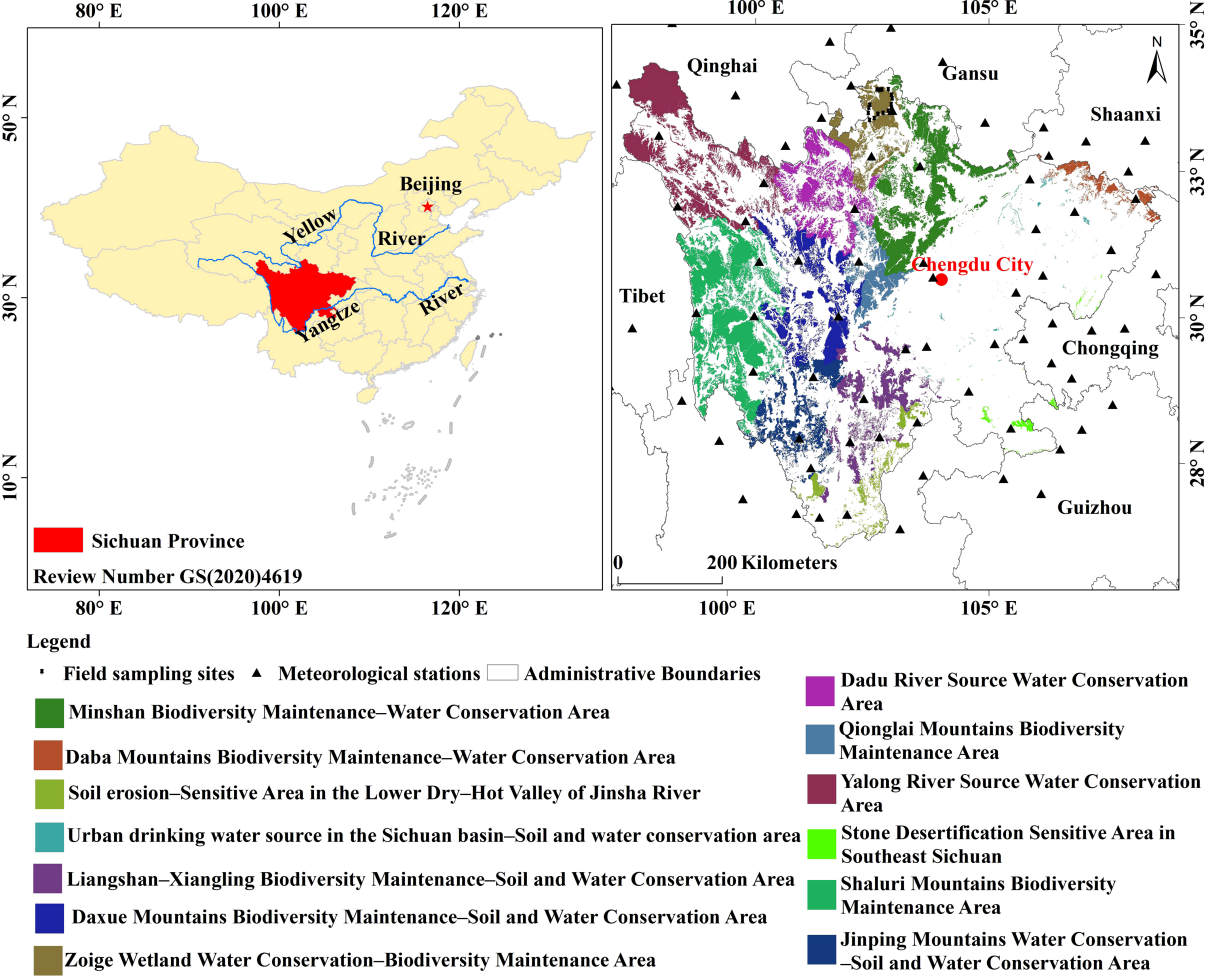
Location of the study area.

Located in the ecological barrier of Qinghai–Tibet Plateau and the Loess Plateau–Chuan–Yunnan ecological barrier areas in the national ecological security strategy pattern, Sichuan Province is a critical water-conservation area in the upper reaches of the Yangtze and Yellow Rivers and a global biodiversity conservation hotspot playing an essential role in water resources protection, ecological environment security, and ecological balance. To strengthen ecological protection, the Sichuan Provincial People’s Government issued a “Notice on the Issuance of the Sichuan Ecological Protection RedLine Program” in 2018. The EPRA is located between latitude 26°05’-34°22’N and longitude 97°34’-108°52’E, with a total area of 14.8×10^4^ km^2^ (**Figure 1**). The region has a complex topography, diverse landforms, rich vegetation, and significant regional climatic differences. The eastern basin of Sichuan belongs to the subtropical humid climate zone (He et al., 2022), with annual average temperatures of 16°C-18°C and annual precipitation of 1000 mm-1300 mm. Northwest Sichuan belongs to the plateau alpine climate zone. It has large altitude differences and noticeable three-dimensional changes in climate, with an annual average temperature of 4°C-12°C and annual precipitation of 500mm-900mm. The mountains of southwest Sichuan belong to the subtropical semi-humid climate zone, with distinct dry and wet conditions, an annual average temperature of 12°C-20°C, an annual precipitation of 900 mm-1200 mm.

### 2.2 Data sources

The research data in this paper include satellite data, meteorological data, soil attribute data, field sampling data, boundary vector data, and afforestation area data. The satellite data mainly include MOD13A1 (500 m, 16-day) vegetation index products (NDVI and enhanced vegetation index (EVI)), MCD12Q1 (500 m) land use data, and MOD16A2GF (500 m, 8-day) evapotranspiration (ET) data from the USGS website [https://earthexplorer.usgs.gov/ (accessed on 22 May 2023)] for 2000-2021. In addition, we employed 30 m digital elevation model data (DEM, ASTERGDEMv3 was released by NASA and METI on August 5, 2019) downloaded from the Geospatial Data Cloud website [https://earthexplorer.usgs.gov/ (accessed on 22 May 2023)]. Pre-processing of MOD13A1 data included reprojection, quality control, reconstruction with S-G filtering, and synthesized as monthly data with maximum values. Pre-processing of MCD12Q1 data included reprojection and extraction of IGBP classification layers. Meteorological data (temperature (TEM), precipitation (PRE), sunshine hours, wind speed (WS)) were obtained from Sichuan Meteorological Bureau. This paper uses the inverse distance weighting method based on DEM correction to interpolate them spatially. Surface solar radiation (SSR) data were estimated by using the Ångström-Prescott model based on sunshine hours (Wang et al., 2014). Soil moisture (SM) data were downloaded from the TerraClimate dataset at ~4 km (1/24th degree) spatial resolution [https://www.climatologylab.org/ (accessed on 22 May 2023)]. Soil attribute data were obtained from the Resource and Environment Science Data Center of the Chinese Academy of Sciences [http://www.resdc.cn (accessed on 22 May 2023)]. All data were processed as the Albers equal-area projections with a spatial resolution of 500 m.

A field biomass survey was conducted using a five-point cross-sampling method. The area of large sample plots was set to 100 m×100 m, and one 1 m×1 m small sample square was set at each corner and center of each sample plot. Biomass samples were obtained by the harvesting method. They were then returned to the laboratory and baked to constant weight at 80°C. The dry matter ratio of the samples was calculated; the total dry weight of above-ground biomass in the sample plots was calculated based on the fresh weight of the sample plots. The root-to-crown ratio was considered 4.25 (Piao et al., 2004); the above-ground and below-ground biomass carbon conversion coefficients were 36.98% and 42.91%, respectively (Chen et al., 2012; Bao et al., 2016). Vegetation NPP was converted based on these parameters and used to validate the CASA (Carnegie–Ames–Stanford approach) model. Additionally, National Nature Reserve data were obtained from the Resource and Environment Science Data Center of the Chinese Academy of Sciences (http://www.resdc.cn (accessed on 22 May 2023)). Afforestation area data were obtained from the Sichuan Statistical Yearbook [http://tjj.sc.gov.cn/scstjj/c105855/nj.shtml (accessed on 22 May 2023)].

### 2.3 Vegetation ecological quality evaluation method

#### 2.3.1 Estimation of ecological functions

This paper selected four ecosystem functions: carbon sequestration, water conservation, soil retention, and biodiversity maintenance. The carbon sequestration function is represented by NPP and was estimated using the CASA model (Cui et al., 2020) (the simulation results of the CASA model were validated by using the field survey data from 2021 (Zoige wetland area) with R^2^ = 0.67 (99% confidence level)). The water balance method estimated the volume of conserved water used to express the water conservation function (WC) (Xu et al., 2022). The modified universal soil loss equation calculated the soil retention function (SR) (Pinson and AuBuchon, 2023). The biodiversity maintenance function is expressed as habitat quality (HQ) and was estimated using the INVEST model (Zhao et al., 2022). **Table S1** shows the detailed calculation equations, data sources, and references, and **Tables S2-S4** shows the input parameters of each model. The four ecosystem functions were normalized using the maximum and minimum values of the respective 2000-2021 time-series data for the three ecological functions. Weights were then calculated using the CRITIC method (Diakoulaki et al., 1995), and the weighted sums were calculated to obtain the ecosystem function time-series data of the EPRA.


(1) $$EF_{\text{i}}=\frac{EF_{i}-EF_{i,\min}}{EF_{i,\max}-EF_{i,\min}}$$

(2) $$EF_{\text{c}}=\sum_{i=1}^{4}w_{i}\times EF_{i}$$

Here, $EF_{i}$ is the *i*th ecological function; $EF_{i,\min}$ and $EF_{i,m\text{ax}}$ are the minimum and maximum values of the *i*th ecological function, respectively; $w_{i}$ is the weight of the *i*th ecological function; and $EF_{c}$ is the integrated ecological function.

#### 2.3.2 Fractional vegetation cover

The FVC was calculated using the mixed pixel decomposition method based on MODIS monthly EVI data. The calculation formula is as follows (Qian et al., 2020):


(3) $$FVC_{year}=\frac{1}{12}\sum_{i=1}^{12}\frac{EVI_{i}-EVI_{soil}}{EVI_{veg}-EVI_{soil}}$$

where EVI_soil_ and EVI_veg_ are the EVIs of soil and vegetation cover, respectively, calculated considering the minimum and maximum values of the EVI of the study area with 0.5% and 99.5% confidence intervals, and FVC_year_ is the average annual vegetation cover.

#### 2.3.3 VEQI

The VEQI is based on EFc and FVC and was calculated as follows:


(4) $$VEQI=\left(FVC\times f_{1}+EF_{\text{c}}\times f_{2}\right)\times 100\%$$

where VEQI is the vegetation ecological quality index, varying between 0 and 100%; FVC is the fractional vegetation cover; and f_1_ and f_2_ are weighting coefficients obtained using the CRITIC method (Diakoulaki et al., 1995).

### 2.4 Trend analysis methods

This paper used the Theil–Sen median trend analysis (Sen slope estimation), Mann–Kendall nonparametric test ( |Z| ), and Hurst index (HI) methods to study the spatial and temporal variation and sustainability characteristics of VEQ in the EPRA. Sen slope estimation was used for trend discrimination (Lu et al., 2020); the Mann–Kendall method was used for the trend significance test (Qi et al., 2019); and the Hurst index was used for trend sustainability judgment (Yuan et al., 2013; Emamian et al., 2021). The future trends of the VEQI were divided into six categories, as shown in **Table 1**.

**Table 1:** Future trend types of the VEQI.

Sen Slope	|Z|	HI	Type	Code
——	≤ 1.96	≤ 0.5	Uncertain	1
< 0	> 1.96	≤ 0.5	Decreasing to increasing	2
> 0	> 1.96	≤ 0.5	increasing to decreasing	3
——	≤ 1.96	> 0.5	Persistently stable	4
< 0	> 1.96	> 0.5	Persistently decreasing	5
> 0	> 1.96	> 0.5	Persistently increasing	6

### 2.5 Contribution rate analysis methods

#### 2.5.1 Residual analysis

Residual analysis (Qi et al., 2019) was used to study the effects and relative contributions of climate change and human activities. It is assumed that the vegetation in the core area of the National Nature Reserve is only affected by climate change. The VEQI of the region was used as the output variable and the climate factors (TEM, PRE, ET, WS, SM, SSR) as the input variables to simulate the VEQI influenced only by climate (VEQIc) using the XGBoost model. A total of 132,008 samples (50% of training and 50% of test samples) were used as input data for XGBoost, and the parameters were tuned using the grid search method (learning_rate=0.3, max_depth=8, n_estimators=1000). The XGBoost model performed well (R^2^ = 0.90, RMSE = 0.09). The relative importance scores were ranked as TEM (0.73) > WS (0.083) > SM (0.078) > SSR (0.042) > ET (0.039) > PRE (0.03). This indicates that VEQI has a higher sensitivity to temperature relative to other climate factors.

Then, the difference (VEQI_H_) between VEQI and VEQI_C_ was calculated to represent the impact of human activities on the VEQI. The specific calculation equation is as follows:


(5) $$VEQI_{H}=VEQI-VEQI_{C}$$

where VEQI_C_ and VEQI_H_ refer to VEQI influenced by climate change and human activities, respectively.

#### 2.5.2 Determination of drivers and calculation of relative contribution

The Sen slope values of VEQI from 2000 to 2021 represented the trends of VEQI changes. A positive trend indicates that the influence factor promotes the increase in the VEQI, while a negative trend indicates that the influence factor inhibits the VEQI. Seven impact levels according to the Sen slope values and |Z| were considered: significantly inhibited, moderately inhibited, slightly inhibited, no effects, slightly promoted, moderately promoted, and significantly promoted (**Table 2**). Additionally, the main drivers of VEQI changes were distinguished based on the trend of VEQI, VEQI_C_, and VEQI_H_, and the relative contributions of climate change and human activities to VEQI changes were calculated (**Table 3**).

**Table 2:** Classification of the effects of climate change and human activities on the VEQ.

Sen Slope	|Z|	Degree of Influence
> 0	> 1.96	Significantly promoted
	1.645~1.96	Moderately promoted
	0~1.645	Slightly promoted
0	——	No effects
< 0	0~1.645	Slightly inhibited
	1.645~1.96	Moderately inhibited
	> 1.96	Significantly inhibited

**Table 3:** Determination of drivers and calculation of their relative contribution rates.

Sen (VEQI)	Driving Factors	Sen Slope		Relative Contribution Rate (%)	
		VEQI_C_	VEQI_H_	Climate Change	Human Activities
>0	C&H	>0	>0	$\frac{Sen\left(VEQI_{C}\right)}{Sen\left(VEQI_{C}\right)+Sen\left(VEQI_{H}\right)}$	$\frac{Sen\left(VEQI_{H}\right)}{Sen\left(VEQI_{C}\right)+Sen\left(VEQI_{H}\right)}$
	C	>0	<0	100	0
	H	<0	>0	0	100
<0	C&H	<0	<0	$\frac{Sen\left(VEQI_{C}\right)}{Sen\left(VEQI_{C}\right)+Sen\left(VEQI_{H}\right)}$	$\frac{Sen\left(VEQI_{H}\right)}{Sen\left(VEQI_{C}\right)+Sen\left(VEQI_{H}\right)}$
	C	<0	>0	100	0
	H	>0	<0	0	100

C refers to climate change; H refers to human activities.

**Figure 2:**
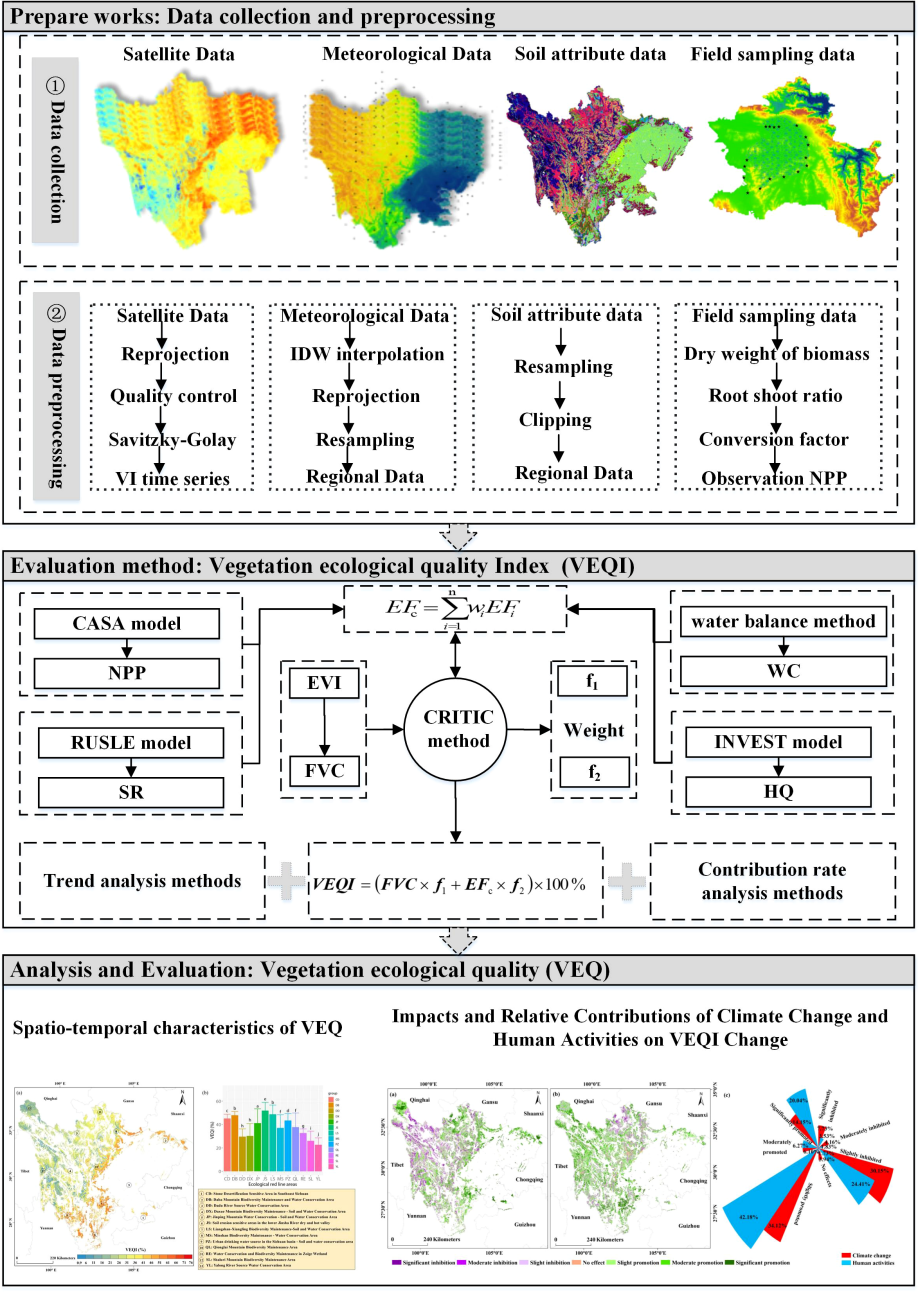
Flow chart of VEQ evaluation.

**Figure 2** displays the research idea and VEQ evaluation system proposed in this paper. The system is divided into three major parts: (1) Data collection, including remote sensing data, meteorological data, soil attribute data, and field survey data, and their pre-processing. (2) Construction of the VEQI: First, the CASA model, the water balance method, and the InVEST model are employed to estimate NPP, WC, SR, and HQ; then, after normalization and weighted summation to obtain the integrated ecological function (EFc), and the EVI is used to estimate the FVC; finally, the CRITIC method is used to calculate the weights of FVC and EFc and to construct the VEQI using weighted summation. (3) Based on the first two steps, trend analysis and the residual analysis based on the XGBoost model are used to study the spatial and temporal variation characteristics of VEQ and the relative contribution of its influencing factors.

## 3 Results

### 3.1 Analysis of changes in VEQI

#### 3.1.1 Spatial patterns of VEQI

**Figure 3:**
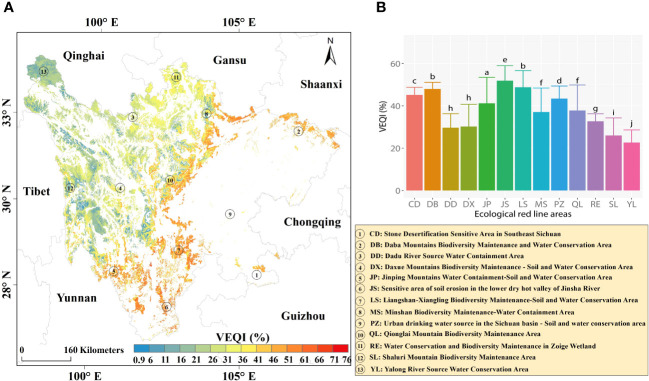
Annual average values of the VEQI from 2000 to 2021: **(A)** annual average values; **(B)** variability between regions.

The multi-year mean values of the VEQI in the EPRA ranged from 0.92% to 75.99%. The VEQI was a decreasing trend from southeast to northwest (**Figure 3A**), with noticeable zonal spatial distribution characteristics. The VEQI of the 13 sub-regions also showed significant differences (**Figure 3B**). The area with the highest mean value of the VEQI was the Soil Erosion–Sensitive Area in the Lower Dry–Hot Valley of Jinsha River (VEQI=51.91%). In contrast, the area with the lowest value was Yalong River Source Water Conservation Area (VEQI=22.68%).

#### 3.1.2 Temporal trends of VEQI

**Figure 4:**
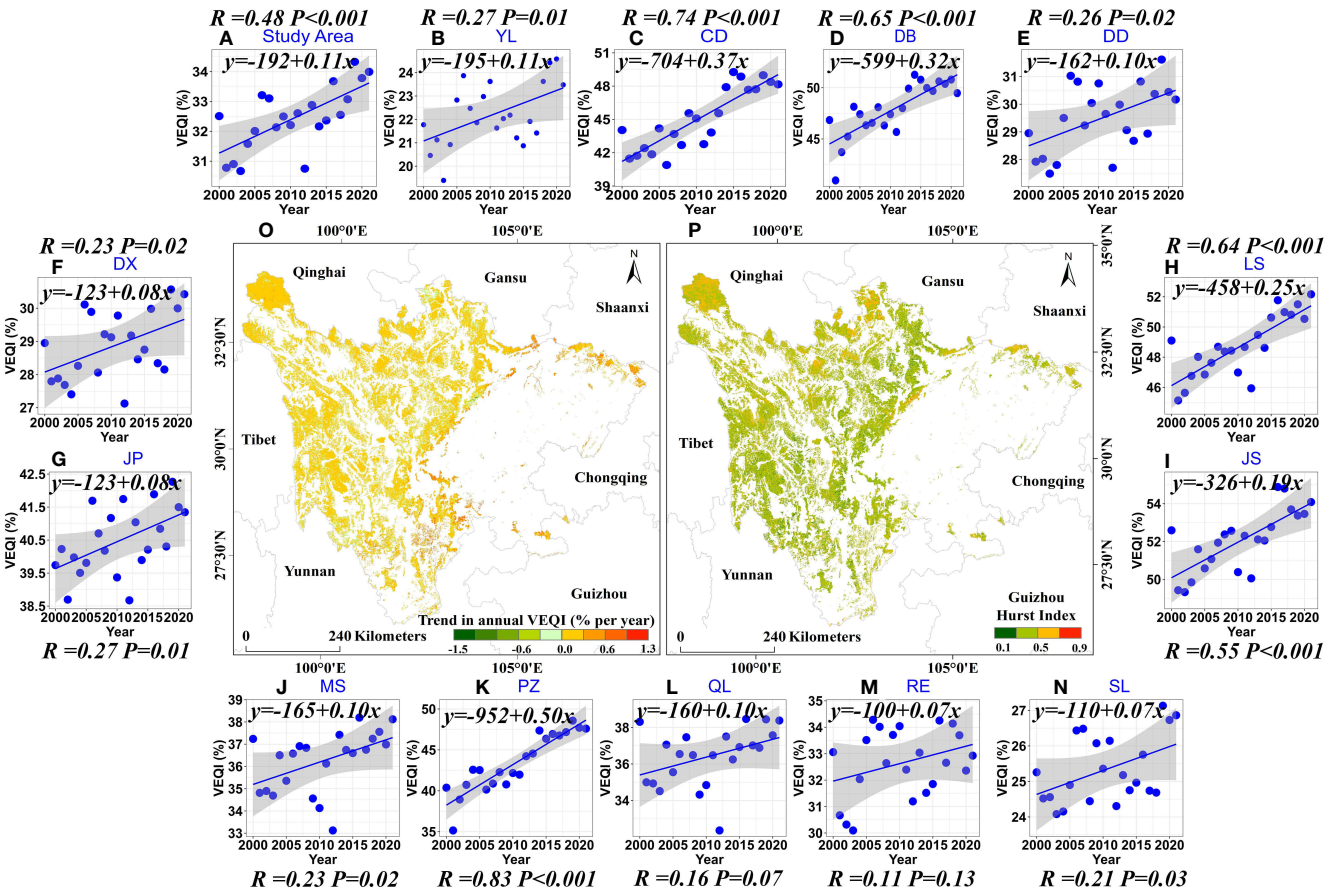
Spatial distribution of VEQI trends from 2000 to 2021 (The abbreviations in the Figure correspond to **Figure 3**).

The interannual variation of VEQI in the EPRA showed a significant upward trend from 2000 to 2021, with a rate of increase of 0.11% year^-1^ (**Figure 4A**). Among the 13 sub-regions, Dadu River Source Water Conservation Area, Zoige Wetland Water Conservation–Biodiversity Maintenance Area, and Shaluli Mountains Biodiversity Area showed a non-significant upward trend, while the other ten areas showed a significantly increasing trend (**Figures 4B–N**). However, there was spatial heterogeneity in the trends of VEQI (**Figure 4O**). The percentage with an upward trend in the VEQI was 82.71% (the area percentage with a significant increase was 27.87%), with the main contributions being those of grassland ecosystems (48.96%) and forest ecosystems (34.41%), mainly in Liangshan–Xiangling Biodiversity Maintenance–Soil and Water Conservation Area, Daba Mountains Biodiversity Maintenance–Water Conservation Area, and Stone Desertification Sensitive area in Southeast Sichuan. In contrast, only 14.02% of the areas showed a decreasing trend (0.86% showed a significant decrease), mainly in the EPRA edges. It can be seen that a recovery trend characterized the vegetation in most of the EPRA.

Future VEQI change trends after 2021 were also examined using the Hurst index (**Figure 4P**). The average Hurst index of the VEQI in the EPRA was 0.44, only 24.68% of the EPRA had a Hurst index above 0.5. Simultaneously, the percentages of the EPRA with VEQIs showing an uncertain trend and a changing trend from increasing to decreasing were 49.36% and 25.97%, respectively, while the area percentages showing a persistent increase were only 9.89%. Moreover, the percentages of the 13 sub-regions with VEQIs showing uncertain trends and change trends from increasing to decreasing in the future ranged from 54.68% to 86.55%. These results indicate that in the future, based on maintaining the existing vegetation restoration measures, more attention should be paid to the ecological status of vegetation in areas where the trend is uncertain and where there is a shift from an upward trend to a downward one.

### 3.2 Impacts and relative contributions of climate change and human activities on VEQI change

#### 3.2.1 Impacts of climate change and human activities on VEQI change

**Figure 5:**
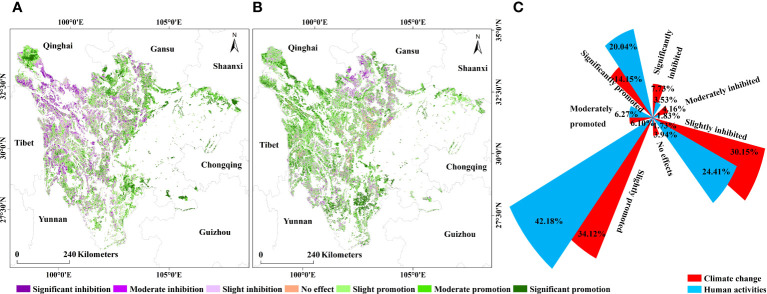
Impact of climate change and human activities on the VEQ from 2000 to 2021: **(A)** climate change; **(B)** human activities; **(C)** the percentage of area.

The effects of climate change and human activities on the VEQI showed evident spatial heterogeneity (**Figures 5A, B**). The area where climate change played a facilitating role in the improvement of VEQ accounted for about 54.37%, among which the area playing a moderate and significant facilitating role accounted for 20.25% (**Figure 5C**), mainly located in the northwestern part of Yalong River Source Water Conservation Area, Zoige Wetland Water Conservation–Biodiversity Conservation Area, the northern part of Liangshan–Xiangling Biodiversity Maintenance–Soil and Water Conservation Area, the eastern part of Daba Mountains Biodiversity Maintenance–Water Conservation Area, Stone Desertification Sensitive area in Southeast Sichuan, and the southeastern part of Minshan Biodiversity Conservation–Water Conservation Area. About 3.94% in the EPRA showed no significant effects of climate change on the change of vegetation ecological quality. The area where climate change inhibited the improvement of VEQ was 41.69%, and the area where its inhibition was moderate and significant was 11.53%, mainly distributed in the central and southern part of Yalong River Source Water Conservation Area, the western and southern part of Shaluli Mountains Biodiversity Maintenance Area, the central and northern part of Daxueshan Biodiversity Maintenance–Soil and Water Conservation Area, and the northern part of Minshan Biodiversity Conservation–Water Conservation Area.

The percentage of areas with no significant effects of human activities on the change in VEQ was 1.73% (**Figure 5C**). Compared with the effects of climate change, the proportion of areas where human activities promoted the improvement of VEQ was larger (68.49%). Human activities significantly promoted the improvement of VEQ in Liangshan–Xiangling Biodiversity Maintenance–Soil and Water Conservation Area, Daba Mountains Biodiversity Maintenance–Water Conservation Area, and Yalong River Source Water Conservation Area. The percentage of areas where human activities had an inhibitory effect on improving VEQ was 29.78%, mainly in Zoige Wetland Water Conservation–Biodiversity Conservation Area, Minshan Biodiversity Conservation–Water Conservation Area, and the northeast part of Dadu River Source Water Conservation Area.

**Figure 6:**
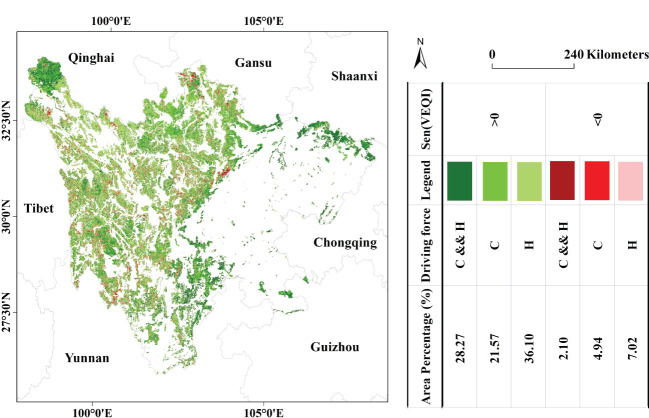
Spatial distribution of drivers of change in VEQI from 2000 to 2021.

About 28.27% in the EPRA showed that climate change and human activities influenced the improvement of the VEQ. The area showing VEQ improvement induced by climate change alone accounted for about 21.57%, mainly in Zoige Wetland Water Conservation–Biodiversity Maintenance Area, the northern part of Liangshan–Xiangling Biodiversity Maintenance–Soil and Water Conservation Area, and Minshan Biodiversity Conservation–Water Conservation Area, while the area showing VEQ improvement induced by human activities alone accounted for 36.10%, mainly in Shaluli Mountains Biodiversity Maintenance Area, Yalong River Source Water Conservation Area, and Dadu River Source Water Conservation Area. In addition, about 2.10% of the area showed that the combined effect of climate change and human activities was the driving factor in the deterioration of VEQ, mainly in Zoige Wetland Water Conservation–Biodiversity Maintenance Area and Minshan Biodiversity Maintenance–Water Conservation Area. The areas showing VEQ deterioration caused by either climate change or human activities alone accounted for 4.94% and 7.02%, respectively, and were relatively scattered (**Figure 6**). Overall, climate change and human activities have been the main reason for the VEQ changes in the EPRA over the past 22 years.

#### 3.2.2 Relative contributions of climate change and human activities to VEQI Change

**Figure 7:**
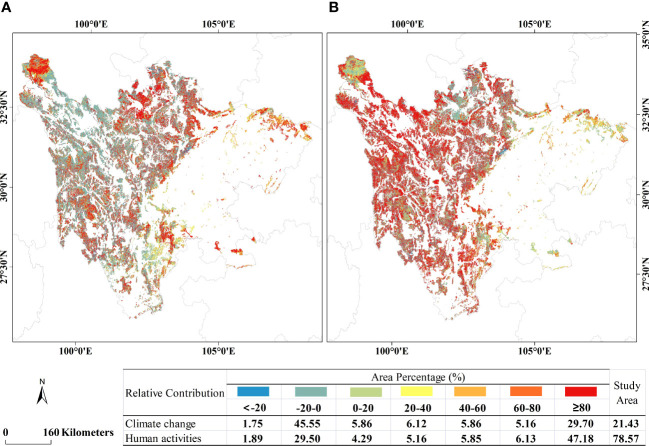
Spatial distribution of the relative contribution of factors influencing VEQ from 2000 to 2021: **(A)** climate change; **(B)** human activities.

The area percentage showing a positive contribution of climate change to the change in VEQI was 52.70% (**Figure 7A**). The area with contribution rates of 20%-40%, 40%-60%, and 60%-80% all accounted for more than 5%. The area with contribution rates greater than 80% accounted for about 29.70%, mainly in the northwestern part of Yalong River Source Water Conservation Area, Zoige Wetland Water Conservation–Biodiversity Conservation Area, the northern part of Liangshan–Xiangling Biodiversity Maintenance–Soil and Water Conservation Area, and the southeastern part of Minshan Biodiversity Conservation–Water Conservation Area. The area percentage showing a negative contribution rate of climate change to the change in VEQI was about 47.30%, mainly in the central and southern part of Yalong River Source Water Conservation Area, Shaluli Mountains Biodiversity Maintenance Area, Qionglai Mountains Biodiversity Maintenance Area, and Daxueshan Biodiversity Maintenance–Soil and Water Conservation Area.

The area percentage showing a positive contribution of human activities to the VEQI change was 68.61% (**Figure 7B**). The percentage of areas with contribution rates of 20%-40%, 40%-60%, 60%-80%, and 80%-100% exceeded 5%. The areas where the contribution was over 80% were mainly in Liangshan–Xiangling Biodiversity Maintenance–Soil and Water Conservation Area, Shaluli Mountains Biodiversity Area, Dadu River Source Water Conservation Area, and Yalong River Source Water Conservation Area. The percentage of areas where the contribution of human activities to the VEQI change was negative was 31.39%, mainly in Zoige Wetland Water Conservation–Biodiversity Conservation Area, Minshan Biodiversity Conservation–Water Conservation Area, and the northeast part of Dadu River Source Water Conservation Area. In most areas, the contribution of human activities to improving VEQ was generally more remarkable than climate change. Overall, the relative contributions of climate change and human activities to VEQI changes in the EPRA were 21.43% and 78.57%, respectively.

## 4 Conclusion

Based on multi-source data, this study developed the VEQI and the residual analysis based on the XGBoost model, and constructed a VEQ evaluation system. The Spatio-temporal patterns, future trends, and the relative contributions of the driving factors were then evaluated and analyzed. We found that the VEQ of the EPRA has improved significantly. Significantly improved areas were located in Liangshan–Xiangling Biodiversity Maintenance–Soil and Water Conservation Area, Daba Mountains Biodiversity Maintenance–Water Conservation Area, and Stone Desertification Sensitive area in Southeast Sichuan. Severe deterioration areas were more dispersed and mainly distributed in the EPRA edges. The Hurst index indicated that the future trend of VEQ changes is unsustainable. Relative to other climate factors, TEM had the greatest impact on VEQ across the EPRA. Climate change and human activities jointly influenced the changes of VEQ. Human activities were the main influence factor with a relative contribution of 78.57% (the relative contribution of climate change was 21.43%).

## 5 Discussion

### 5.1 Reliability of indicators and methods

**Figure 8:**
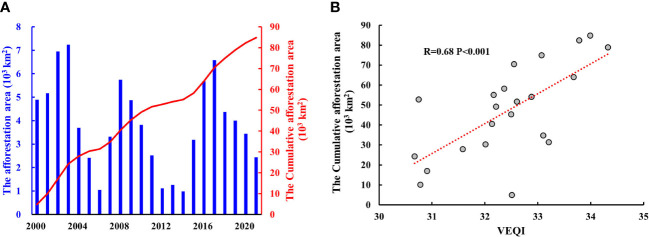
Afforestation area and its correlation with VEQI: **(A)** afforestation area; **(B)** correlation of VEQI with cumulative afforestation area.

The reliability of indicators and methods directly affects the accuracy of results. The afforestation and ecological restoration projects control soil erosion and significantly enhance VEQ (Li C. et al., 2022). we analyzed the correlation between cumulative afforestation area and VEQI based on the statistical yearbook data. Cumulative afforestation area in Sichuan Province from 2000 to 2021 was 8.48×10^4^ km^2^, and was significantly correlated with VEQI with a correlation coefficient of 0.68 (**Figure 8**). This proves the reliability of the VEQI proposed in this paper. The reason why the correlation coefficient is not particularly high may be related to the predominantly grassland surface cover in some areas (e.g. Zoige Wetland Water Conservation-Biodiversity Maintenance Area, Yalong River Source Water Conservation Area and Dadu River Source Water Conservation Area). Meanwhile, we compared the applicability of XGBoost and stepwise regression models. The R^2^ of XGBoost improved by 0.13 compared to the stepwise regression model (R^2^ = 0.77), which indicates that the XGBoost model has more explanatory power and the results obtained are more reliable.

### 5.2 Results interpretability

#### 5.2.1 Spatio-temporal characteristics in VEQ

In this study, we used time-series remote sensing data to obtain information on the VEQ in the EPRA from 2000 to 2021. The VEQ showed a spatially decreasing trend from southeast to northwest with noticeable zonal spatial distribution characteristics. The highest-value and lowest-value areas were located in Soil Erosion–Sensitive Area in the Lower Dry–Hot Valley of Jinsha River and Yalong River Source Water Conservation Area, respectively. Li G. et al. (2022) reached the same conclusion when studying the spatial distribution characteristics of vegetation NPP in western Sichuan. The main reason for this may be that the primary vegetation type in the southeastern part of the EPRA is woodland, which has higher vegetation cover and productivity. In contrast, the northwestern part is mostly grassland, with relatively lower vegetation cover and productivity.

Similar to other studies (Macias-Fauria, 2018; Ge et al., 2021; Pei et al., 2022), our study also revealed the VEQ in the EPRA has improved to some extent over the past two decades. The areas with increasing and decreasing trends of the VEQI accounted for 82.71% and 14.02% of the total area of the EPRA, respectively, which is also consistent with the findings obtained by Li S. et al., (2022). However, there are differences in the proportion of areas showing significant increasing and decreasing trends due to differences in study periods, data, and analysis methods. Taking Zoige Wetland Water Conservation–Biodiversity Maintenance Area as an example, this study showed a non-significantly increasing trend of the VEQI in this area, consistent with the results obtained by Shen et al. (2019). At the same time, there was also significant spatial heterogeneity: the area percentages with increasing and decreasing trends of the VEQI were 80.08% and 19.92%, respectively (**Figure 4A**). Feng et al. (2015) analyzed the spatial and temporal characteristics of vegetation changes in the Zoige region from 2000 to 2013. They pointed out that after 2008, the VEQ has improved in some places, while it has continued to deteriorate in others. In addition, according to the Hurst index, we found that the trend of the VEQI in the EPRA could be unsustainable in most areas, which indicates that the relevant authorities should pay continuous attention to vegetation conservation and ecological construction in the region.

#### 5.2.2 Influencing factors of VEQ

The regions where climate change and human activities contribute to the VEQ exceeding 80% are different. The areas where human activities contribute more than 80% are located in Liangshan–Xiangling Biodiversity Maintenance–Soil and Water Conservation Area, Daba Mountains Biodiversity Maintenance–Water Conservation Area, and Yalong River Source Water Conservation Area, which may be mainly due to the implementation of the Grain to Green Program since 1999 (Jiang et al., 2020). As of 2019, the area returned to forest and grassland in Sichuan Province was about 26,700 km^2^, and the forest vegetation cover had increased by about 4% and locally by more than 10%. The results of this study showed a significant upward trend in VEQ from 2000 to 2021, with an increased rate of 0.11% (**Figure 4A**) and a maximum increase rate of 1.3% (**Figure 4O**). The regions where the contribution of human activities exceeds 80% are all in the priority implementation areas for returning farmland to forest and grassland. This result is consistent with the conclusion of Zheng et al. (2019) and Jin et al. (2019) that “human activities effectively contribute to vegetation restoration at local or regional scales.” We quantified the relative role of human activities on VEQI change in the EPRA using the residual analysis based on the XGBoost model, and found that the vast majority of VEQ improvement was induced by human activities. Consistent with our results, a growing number of studies have also highlighted the existence of positive effects of human activities, especially large-scale afforestation and ecological restoration projects, on vegetation recovery (Zhao et al., 2018; Niu et al., 2019; Zhao et al., 2020; Pei et al., 2022).

Climate change is also crucial to vegetation dynamics (Liu et al., 2018; Ge et al., 2021). Numerous studies have shown that precipitation and temperature are the most important climatic factors affecting vegetation growth (Luo et al., 2018; Teng et al., 2020). The areas with more than 80% contribution to climate change are mainly located in the northwestern part of Yalong River Source Water Conservation Area, Zoige Wetland Water Conservation–Biodiversity Conservation Area, the northern part of Liangshan–Xiangling Biodiversity Maintenance–Soil and Water Conservation Area, and the southeastern part of Minshan Biodiversity Conservation–Water Conservation Area, which may be mainly because these places are nature reserves, and ecological restoration is based chiefly on natural restoration and supplemented by artificial restoration (Wu et al., 2022). The climate in the EPRA shows warming and wetting trends (Zhang et al., 2022). The warming and humid climate favors vegetation growth, with a significant increase in vegetation cover and NPP and a significant improvement in VEQ (Wei et al., 2022). According to importance scores of variables, our findings seem to support what previous studies have concluded: “vegetation growth in Sichuan Province is more influenced by temperature than precipitation (Fang et al., 2002)”. However, higher temperatures may lead to earlier phenological periods and longer growing seasons (Xiong et al., 2021), enhance vegetation photosynthetic efficiency (Xu et al., 2013), and accelerate soil organic matter decomposition and nutrient release (Li et al., 2017), thus favoring the improvement in VEQ.

Climate change and human activities can also inhibit the improvement in the VEQ and even lead to the degradation of the VEQ. On the one hand, the continuous increase in temperature may directly or indirectly affect plant transpiration and soil evapotranspiration (Jia et al., 2022). On the other hand, the increase in precipitation reduces solar radiation, inhibits photosynthesis, and increases soil water content, which puts the soil in an anaerobic state (Yang et al., 2015), which is unfavorable to the improvement of the VEQ. Unchecked human activities, such as overgrazing, can also decrease VEQ (Guo et al., 2021).

### 5.3 Implications for vegetation ecological conservation and management

Although the overall trend of VEQI in the EPRA was significantly increasing, the trend is unsustainable in most areas according to the Hurst index analysis. This result suggests that the already fragile vegetation ecosystems are even more unstable. At the same time, we found the relative contribution from human activities to be very significant. Therefore, changes in VEQ will require more attention and sustained input.

The warm and humid climate trend in the EPRA is favorable to improving the VEQ. However, the long-term temperature increase may be detrimental to the growth of coniferous forests (Liu et al., 2018). Studies have shown that the intensity, duration, and frequency of seasonal drought events in Sichuan Province, China are also increasing (Yu et al., 2014), and extreme temperatures pose a severe threat to VEQ (Barriopedro et al., 2012; Cao et al., 2022), which may offset the effectiveness of ecological restoration projects for VEQ improvement. Therefore, under future climate change scenarios, special attention should be paid to more targeted measures to cope with the effects of extreme climate events on the VEQ. For example, it is recommended to prioritize using drought- and heat-tolerant tree species in future afforestation projects.

### 4.4 Shortcomings and prospects

Limitations still exist in this study. Firstly, this study reduces the climatic factors affecting the changes in VEQ to temperature and precipitation, radiation, evaporation, wind speed and soil moisture, and attributes all the remaining unnatural factors to human activities. However, numerous studies have shown that many other factors, such as nitrogen deposition, atmospheric CO_2_ concentration, fire, and soil properties, influence vegetation changes. Second, the time-lag effect of climatic factors on vegetation growth should have been considered. In addition we do not consider the complex interactions between climate change and human activities.

Given the above shortcomings, the selection of influence factors needs to be more comprehensive. Meanwhile, more robust methods must be proposed to separate the effects of climate change and human activities. The time-lag effect and complex interactions of the influence factors also needs to be considered to more accurately quantify the contributions of the influencing factors to VEQ changes.

## Data availability statement

The original contributions presented in the study are included in the article/**Supplementary Material**. Further inquiries can be directed to the corresponding authors.

## Author contributions

CL: Conceptualization, Methodology, Investigation, Funding acquisition, Writing – original draft preparation. CY: Supervision, Resources – review & editing. LG and YY: Resources – review & editing. LY: Data curation, Resources – review & editing. HS: Data curation – review & editing. All authors contributed to the article and approved the submitted version.
