# Supplementary material for: Comprehensive evaluation system for vegetation ecological quality: a case study of Sichuan ecological protection redline areas
Source: Front Plant Sci. 2023 Jun 26;14:1178485. doi: 10.3389/fpls.2023.1178485 (PMC10331475; doi:10.3389/fpls.2023.1178485)
Supplement: Supplementary file 1 [file Table_1.docx]

Supporting Information for

**Supporting Information Ⅰ: Tables**

**Table S1.** Date sources and estimation methods of four EFs

| EFs | Estimation Method | Input Parameters | Datasets and reference |
| --- | --- | --- | --- |
| Water Containment | Based on the water balance method, WC is estimated as follow,  $WC=(Pre-Runoff-AET)\times A$  $WC$: the volume of the water containment (m^3^),  $P\mathrm{re}$: the annual precipitation(mm),  $Runoff$: the Surface runoff (mm),  $AET$: the actual evapotranspiration (mm),  $A$: the regional or pixel area (m^2^).  The surface runoff is calculated as follow,  $Runoff=Pre\times\alpha$  $\alpha$: the coefficient of surface runoff. | Land use data (MCD12Q1)  Actual evapotranspiration data (MOD16A2GF) | USGS website https://earthexplorer.usgs.gov/ |
|  |  | surface runoff coefficient | (Gong et al., 2017;  Hu et al., 2018;  Yuan et al., 2020) |
|  |  | Precipitation data | Sichuan Meteorological Bureau |
| Soil retention | The soil retention (SR) is calculated as follow (Fu et al., 2011; He et al., 2019),  $SR=R\times K\times L\times S\times\left( 1-C\times P \right)$  $R=\sum_{i=1}^{12} 1.735\times{10}^{(1.5{log}_{10}\frac{{Pre}_{i}^{2}}{Pre}-0.08188)}$  $K=0.1317\times\left\{ 0.2+0.3{exp}^{\left[ -0.0256SAN\left( 1-\frac{SIL}{100} \right) \right]} \right\}\times\left( \frac{SIL}{CLA+SIL} \right)^{0.3}\times\left( 1-0.25\times\frac{C_{org}}{C_{org}+{exp}^{3.72-2.95C_{org}}} \right)\times\left( 1-\frac{0.7\times{SN}_{a}}{{SN}_{a}+{exp}^{-5.51+22.9{SN}_{a}}} \right)$  ${SN}_{a}=1-\frac{SAN}{100} L=\left( \frac{\beta}{22.13} \right)^{m} \beta=\frac{DEM}{sin\left( \theta\right)}$  $m=\left\{ \begin{aligned} 0.5 \theta\geq9 \\ 0.4 9>\theta\geq3 \\ 0.3 3>\theta\geq1 \\ 0.2 \theta<1 \end{aligned} \right.$  $S=\left\{ \begin{aligned} 10.8\times sin\left( \theta\right)+0.03 \theta<9 \\ 16.8\times sin\left( \theta\right)-0.05 \theta\geq9 \end{aligned} \right.$  $C=\left\{ \begin{aligned} 1 FVC=0 \\ 0.6508-0.3436\times ln\left( FVC \right) 0<FVC\leq0.783 \\ 0 FVC>0.783 \end{aligned} \right.$  $P=0.2+0.03\gamma$  $R$: the rainfall-runoff erosivity factor (MJ·mm/(ha·h)),  ${Pre}_{i}$: the monthly mean precipitation (mm),  $Pre$: the annual mean precipitation (mm),  $K$: the soil erodibility factor (t·ha·h/(ha·mm·MJ)),  $SAN$: the percentage of the sand in the soil (%),  $SIL$: the percentage of the silt in the soil (%),  $CLA$:the percentage of the clay in the soil (%),  $C_{org}$: the percentage of the organic carbon in the soil (%),  $L$: the slope length factor,  $S$: the slope gradient factor,  $\beta$: the mean slope length (m),  $m$: the variable changing with the slope,  $\theta$: the mean percent slope (%),  $C$: the vegetation cover factor,  $FVC$: the vegetation coverage,  $P$: the erosion control factor,  $\gamma$: the percentile slope gradient. | Land use data (MCD12Q1) | USGS website https://earthexplorer.usgs.gov/ |
|  |  | MODIS EVI data  (MOD13A1) |  |
|  |  | DEM data |  |
|  |  | Precipitation data | Sichuan Meteorological Bureau |
|  |  | Soil attribute data | Data Center for Resources and Environmental Sciences,Chinese Academy of Sciences http://www.resdc.cn |
| Carbon storage | NPP (net primary productivity) is estimated using the CASA model,  $NPP=\mathrm{SOL}\times0.5\times\mathrm{FPAR}\times\varepsilon$  $\varepsilon=\mathrm{Tg}_{1}\times\mathrm{Tg}_{2}\times W_{g}\times\varepsilon_{\max}$  $SOL$: total solar radiation, estimated using the Ångström-Prescott model based on sunshine hours ($\mathrm{MJ}\cdot m^{-2}\cdot\mathrm{month}^{-1}$),  $FPAR$: the absorption fraction of photosynthetically active radiation by vegetation canopy, calculated using NDVI data,  $\varepsilon$: the actual light use efficiency ($g C\cdot\mathrm{MJ}^{-1}$),  ${Tg}_{1} and {Tg}_{2}$: temperature stress coefficients ($g C\cdot\mathrm{MJ}^{-1}$),  $Wg$: the water stress coefficient,  $\varepsilon_{max}$: the maximum light use efficiency ($g C\cdot\mathrm{MJ}^{-1}$). | Land use data  (MCD12Q1) | USGS website https://earthexplorer.usgs.gov/ |
|  |  | MODIS EVI data  (MOD13A1) |  |
|  |  | Evapotranspiration data (MOD16A2GF) |  |
|  |  | Sunshine hours data | Sichuan Meteorological Bureau |
|  |  | Temperature data |  |
|  |  | Maximum light use efficiency | (Zhu et al., 2007;  Su et al., 2022) |
| Habitat quality | Habitat quality (HQ) is computed as follow,  ${HQ}_{xj}=H_{j}\left( 1-\left( \frac{D_{xj}^{z}}{D_{xj}^{z}+k^{z}} \right) \right)；$  $D_{xj}=\sum_{r=1}^{R} \sum_{y=1}^{Y_{r}} \left( \frac{w_{r}}{\sum_{r=1}^{R} w_{r}} \right)r_{y}i_{rxy}\beta_{x}S_{jr}$  *j*=1, 2, …, n: the *j*th land use type,  *r*=1, 2, …, R: the *r*th threat,  *y*=1, 2, …, Y_r_: the *y*th grid on the *r*th threat data,  *H_j_*: the habitat suitability of the *j*th land use type,  $D_{xj}$: the total threat index of the grid $x$ of the land use $j$,  *w_r_*: the relative weight of the threat factor *r*,  *r_y_*: the value of the threat factor on the grid *y*,  *β_x_*: the threat accessibility of the grid x,  *S_jr_*: the sensitivity of the *j*th land use to the threat factor *r*,  *z*: scaling parameters,  *k*: the half-saturation constant,  *i_rxy_*: the impact coefficient of threat *r* in the grid *y* on the grid *x*. | Land use data (MCD12Q1) | USGS website https://earthexplorer.usgs.gov/ |
|  |  | Sensitivity data | (Terrado et al., 2016; Zahra et al., 2018; Rimal et al., 2019; Zhao et al., 2022) |
|  |  | Threat data |  |

**Table S2.** The average runoff coefficients and maximum light use efficiency of different landscape types

| Land use type | Average runoff coefficient (%) | Maximum light use efficiency ($g C\cdot\mathrm{MJ}^{-1}$) |
| --- | --- | --- |
| Evergreen broadleaf forests | 4.65 | 0.985 |
| Evergreen needleleaf forests | 4.52 | 0.389 |
| Mixed forests | 3.52 | 0.720 |
| Deciduous broadleaf forests | 2.70 | 0.692 |
| Deciduous needleleaf forests | 0.88 | 0.485 |
| Closed shrublands | 4.26 | 0.429 |
| Open shrublands | 19.20 | 0.429 |
| Woody savannas | 3.87 | 0.542 |
| Savannas | 3.94 | 0.542 |
| Grasslands | 8.20 | 0.542 |
| Cropland/Natural vegetation Mosaics | 2.40 | 0.542 |
| Water Bodies | 0.00 | 0.000 |
| Permanent wetlands | 0.00 | 0.542 |
| Barren | 3.31 | 0.542 |
| Croplands | 2.40 | 0.542 |
| Urban and Built-up lands | 75.00 | 0.542 |

**Table S3.** Habitat types and their sensitivity to threats

| Land use type | Habitat suitability | Sensitivity | | | |
| --- | --- | --- | --- | --- | --- |
|  |  | Cropland/Natural vegetation Mosaics | Barren | Croplands | Urban and Built-up lands |
| Evergreen broadleaf forests | 1 | 0.6 | 0.35 | 0.6 | 0.4 |
| Evergreen needleleaf forests | 1 | 0.6 | 0.35 | 0.6 | 0.4 |
| Mixed forests | 1 | 0.6 | 0.35 | 0.6 | 0.4 |
| Deciduous broadleaf forests | 0.9 | 0.6 | 0.35 | 0.6 | 0.4 |
| Deciduous needleleaf forests | 0.9 | 0.6 | 0.35 | 0.6 | 0.4 |
| Closed shrublands | 0.85 | 0.6 | 0.1 | 0.6 | 0.5 |
| Open shrublands | 0.7 | 0.6 | 0.2 | 0.6 | 0.55 |
| Woody savannas | 0.8 | 0.8 | 0.7 | 0.8 | 0.5 |
| Savannas | 0.7 | 0.8 | 0.75 | 0.8 | 0.65 |
| Grasslands | 0.7 | 0.8 | 0.75 | 0.8 | 0.65 |
| Cropland/Natural vegetation Mosaics | 0.3 | 0 | 0.5 | 0 | 0.6 |
| Water Bodies | 0.8 | 0.7 | 0.6 | 0.7 | 0.7 |
| Permanent wetlands | 0.85 | 0.75 | 0.6 | 0.75 | 0.65 |
| Barren | 0.2 | 0.1 | 0 | 0.1 | 0.7 |
| Croplands | 0.3 | 0 | 0.5 | 0 | 0.6 |
| Urban and Built-up lands | 0 | 0 | 0 | 0 | 0 |

**Table S4.** Threat factors data

| Threat type | Maximum distance of influence (km) | Weight | Decay form |
| --- | --- | --- | --- |
| Cropland/Natural vegetation Mosaics | 1 | 0.3 | linear |
| Barren | 2 | 0.5 | exponential |
| Croplands | 1 | 0.3 | linear |
| Urban and Built-up lands | 8 | 1 | exponential |

References

Fu, B.J., Liu, Y., Lü, Y.H., He, C.S., Zeng, Y., Wu, B.F. (2011). Assessing the soil erosion control service of ecosystems change in the Loess Plateau of China. Ecol. Complex. 8(4), 284-293 https://doi.org/10.1016/j.ecocom.2011.07.003.

Gong, S.H., Xiao, Y., Zeng, H., Xiao, Y., Ouyang, Z.Y. (2017). Spatial patterns of ecosystem water conservation in China and its impact factors analysis. Acta Ecol. Sin. 37(7), 2455-2462 https://doi.org/10.5846/stxb201512012406.

He, S., Zhu, W., Cui, Y., He, C., Ye, L., Feng, X., Zhu, L. (2019). Study on Soil Erosion Characteristics of Qihe Watershed in Taihang Mountains Based on the InVEST Model. Resour. Environ. Yangtze Basin 28(2), 426-439.

Hu, W.H., He, X.H. (2018). Cascaded connection queuing model of urban drainage system layout and its optimization algorithm. J. Tongji Univ. 46(1), 141-146.

Rimal, B., Sharma, R., Kunwar, R., Keshtkar, H., Stork, N. E., Rijal, S., Rahman, S. A., Baral, H. (2019). Effects of land use and land cover change on ecosystem services in the Koshi River Basin, Eastern Nepal. Ecosyst. Serv. 38, 100963 https://doi.org/10.1016/j.ecoser.2019.100963.

Su, S.T., Zeng, Y., Zhao, D., Zheng, Z.J., Wu, X.H. (2022). Optimization of net primary productivity estimation model for terrestrial vegetation in China based on CERN data. Acta Ecol. Sin. 42(4), 1276-1289 https://doi.org/10.5846/stxb202011263031.

Yuan, Y., Zhang, L., Cui, L.L. (2020). Spatiotemporal variations of water conservation capacity in Ruoergai Plateau. Chin. J. Ecol. 39(8), 2713-2723 https://doi.org/10.13292/j.1000-4890.202008.027.

Zahra, A., Abdolrassoul, S., Yousef, S., Seyed, H.M., Himlal, B., Mojgansadat, A. (2018). Dynamic trade-off analysis of multiple ecosystem services under land use change scenarios: Towards putting ecosystem services into planning in Iran. Ecol. Complex. 36, 250-260 https://doi.org/[10.1016/j.ecocom.2018.09.003](https://www.x-mol.com/paperRedirect/1307832463085768704).

Zhao, Y.H., Qu, Z., Zhang, Y., Ao, Y., Han, L., Kang, S.Z., Sun, Y.Y. (2022). Effects of human activity intensity on habitat quality based on nighttime light remote sensing: A case study of Northern Shaanxi, China. Sci. Total Environ. 851, 158037 https://doi.org/10.1016/j.scitotenv.2022.158037.

Zhu, Y.Q., Pan, Y.Z., Zhang, J.S. (2007). Estimation of net primary productivity of Chinese terrestrial vegetation based on remote sensing. J. Plant Ecol. 31(3), 413-424 https://doi.org/.
